# Biochar Nanoparticles Reduce Ciprofloxacin Accumulation and Restore Growth and Hormonal Balance in Rice Seedlings

**DOI:** 10.3390/plants14030380

**Published:** 2025-01-26

**Authors:** Xiaohan Chen, Jieyu Chen, Yanhong Zhang, Chen Ling, Yu Shen

**Affiliations:** 1 Co-Innovation Center for Sustainable Forestry in Southern China, College of Ecology and Environment, National Positioning Observation Station of Hung-tse Lake Wetland Ecosystem in Jiangsu Province, Nanjing 210037, China; cxiaohan2022@163.com (X.C.); cjy20000708@163.com (J.C.); 2 College of Ecology and Environment, Nanjing Forestry University, Nanjing 210037, China; 3 Jiangsu Provincial Key Laboratory of Environmental Engineering, Jiangsu Provincial Academy of Environmental Science, Nanjing 210037, China; njnuzhangyh@163.com

**Keywords:** ciprofloxacin, biochar nanoparticles, phytohormones, stress reduction, rice seedlings

## Abstract

Ciprofloxacin (CIP), a widely used fluoroquinolone antibiotic, poses a growing environmental concern due to its persistence in agricultural soils and potential adverse effects on crop production. While previous studies have documented CIP’s negative impacts on plant growth, effective strategies to protect crops from antibiotic stress remain limited. Biochar-based approaches show promise, but their application at the nanoscale for antibiotic stress management is largely unexplored. This study demonstrates how biochar nanoparticles (BNPs) effectively mitigate CIP-induced stress in rice seedlings through adsorption mechanisms. Rice seedlings were treated with 5 and 10 mg L^−1^ CIP, with and without 0.2 g L^−1^ BNPs. Results showed that CIP significantly disrupted plant growth, decreasing shoot length by 20.5% and root length by 45.2%, along with reduced biomass. Application of BNPs effectively reduced CIP bioavailability by over 80%, leading to a decreased CIP accumulation of 49.7% in shoots and 33.1% in roots. The addition of BNPs mitigated these growth impacts by restoring shoot length to 98.2% of control levels at 5 mg L^−1^ CIP and improving root growth and biomass accumulation. BNPs also mitigated CIP-induced hormone imbalance, evidenced by a recovery in IAA levels by 8.9%, an increase in 6-BA by 152.6%, and an enhancement in SA levels by 12.7–13.6%. These findings demonstrate the significant potential of nanoscale biochar in reducing antibiotic stress in agricultural systems and provide insights into plant responses under these conditions. This research offers a promising strategy for enhancing crop resilience in areas affected by pharmaceutical pollutants.

## 1. Introduction

Antibiotic contamination in agricultural environments has emerged as a significant environmental concern with implications for both food security and ecosystem health. The widespread use of antibiotics, particularly fluoroquinolones (FQs), in human medicine and animal husbandry has led to their accumulation in agricultural soils through multiple pathways, including wastewater irrigation, biosolid application, and animal manure use [1,2]. FQs are among the most widely used drugs globally due to their high antibacterial efficacy, low toxicity, good therapeutic effects, and low allergenic potential. They are extensively employed in treating bacterial infections in both humans and animals [3,4]. While these practices enhance soil fertility and crop yields, they simultaneously create routes for antibiotic persistence in terrestrial environments [5].

**Figure 1:**
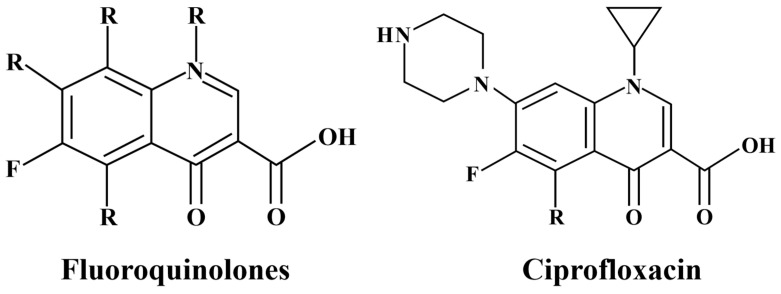
General structure of fluoroquinolones (**left**) and ciprofloxacin (**right**).

FQs pose unique challenges in agricultural systems due to their complex interactions with both soil matrices and plant physiology. The presence of fluorine atoms in their chemical structure, as shown in Figure 1, imparts high electronegativity, stability, and resistance to microbial degradation, leading to environmental persistence issues [6,7]. Once these contaminants accumulate in soil through manure application, wastewater irrigation, and biosolids reuse [8], they may be absorbed by crops, resulting in contamination of agricultural products. The potential environmental hazards of FQs have raised societal concerns regarding food supply safety, potentially compromising the integrity of the “farm-to-folk” supply chain [9]. Studies have shown that FQs also exert negative impacts on plant physiology beyond accumulation. In plants, FQs can inhibit growth, disrupt cellular ultrastructure, and induce oxidative stress, with documented effects in various crops, including rice, wheat, and maize, etc. [7,10].

CIP, a widely used fluoroquinolone antibiotic, poses significant environmental concerns due to its prevalence in soils globally. CIP concentrations as low as 100 μg L^−1^ can trigger oxidative stress in wheat, accompanied by a decline in photosynthetic pigments and assimilation [11]. When maize irrigation water contains ciprofloxacin (0.2 μg L^−1^), morphological and physiological changes occur, leading to a 69% reduction in corn grain yield [12]. At 300 mg L^−1^ in soil, CIP significantly reduces root elongation in lettuce and tomato by over 91% and 99%, respectively, while shoot elongation decreases by 75% and 72%, respectively [13].

To address FQ contamination in agricultural environments, various remediation strategies have been proposed. Biochar-based approaches have shown promise through adsorption mechanisms. For example, seaweed kelp (*Laminaria japonica*) biochar has demonstrated excellent performance in binding CIP in soil, showing approximately 95.06% removal within 180 min while improving cucumber seed germination rates by about 40% [14]. Similarly, zirconium-loaded corn husk biochar has shown effective adsorption of quinolone drugs, with maximum binding capacities of 250–300 mg g^−1^ at pH 3 [15]. Bamboo biochar has also exhibited significant potential in removing FQ antibiotics through surface binding, with removal rates reaching up to 99% [9].

Recent advances in nanotechnology offer promising approaches for addressing FQ contamination in agricultural systems. Biochar nanoparticles (BNPs) have been shown as promising agents for FQ remediation. BNPs offer several advantages over bulk biochar due to their unique properties at the nanoscale. Their extremely high surface-area-to-volume ratio enhances adsorption capacity and reactivity, and their small size allows for better dispersion and potentially increased bioavailability in soil systems. These characteristics make BNPs a particularly promising tool for mitigating antibiotic stress in agricultural environments, as evidenced by studies on carbon nanomaterials and their interactions with FQ antibiotics [16]. In addition, nanofertilizers of BNPs have demonstrated their effectiveness on many crops, including grains, vegetables, and industrial crops, helping to increase growth rates, the concentration of photosynthetic pigments, and productivity [17]. For instance, multi-walled carbon nanotubes (MWCNTs) have proven to be effective adsorbents for removing CIP from aqueous solutions, primarily through electrostatic attractions. Studies have reported maximum adsorption capacities of 1.7446 mg g^−1^ for MWCNTs, with over 88% removal efficiency for CIP concentrations of 4 mg L^−1^ [18]. Nano-graphene materials have also demonstrated significant adsorption capacities for FQ antibiotics, with maximum adsorption capacities for CIP reaching up to 323 mg g^−1^ [19]. The adsorption mechanism is primarily attributed to physical adsorption resulting from non-covalent interactions between the antibiotics and graphene materials.

This study investigates how biochar nanoparticles (BNPs) influence CIP-induced stress responses in rice seedlings through surface binding mechanisms. We hypothesize that BNPs can decrease CIP bioavailability via adsorption, thereby reducing its negative effects on plant growth and hormone balance. Our research examines the quantitative relationships between BNP application, CIP accumulation, plant growth parameters, and endogenous hormone levels in rice seedlings. By investigating the adsorption capabilities of BNPs and their effects on plant physiological responses, this work advances our understanding of nanomaterial applications in addressing emerging agricultural challenges while maintaining transparency about the persistence of bound contaminants in the environment.

## 2. Materials and Methods

### 2.1. Preparation and Characteristics of BNPs

The BNPs used in this study were derived from corn straw collected in Jiangning district, Nanjing, Jiangsu in 2024 in spring. The corn straw biochar was prepared at 450 °C under a nitrogen atmosphere (flow rate: 100 mL min^−1^) with a heating rate of 10 °C min^−1^ and a residence time of 2 h. To remove potential contaminants adsorbed during the combustion process, the biochar underwent thorough washing with ethanol as a solvent prior to further processing. The bulk biochar was then subjected to size reduction using a planetary ball mill (PM 100, Retsch, Germany) operating at 300 rpm for 4 h with zirconia balls (diameter: 5 mm) at a ball-to-material ratio of 10:1. The ground material underwent sequential wet-sieving through 500- and 200-mesh sieves, followed by membrane filtration using polyethersulfone membranes (pore sizes: 450 nm, 220 nm, and 100 nm). Final separation was achieved through differential centrifugation (30,000× *g*, 2 h, 4 °C), yielding approximately 8.5% of the initial biochar mass as nanoparticles. The surface morphology and microstructure of the prepared samples were examined via transmission electron microscopy (TEM) (JEM-1400, Jeol Ltd., Tokyo, Japan), confirming a mean particle diameter of 50 ± 5 nm with a polydispersity index of 0.21. X-ray diffraction (XRD) (Ultima IV, Rigaku, TX, USA) was employed to study the crystal characteristics of the prepared BNPs using Cu Kα as the radiation source with a scanning scope of 2θ = 5–60° and a scan rate of 5° min^−1^. Additionally, Fourier transform infrared (FT-IR) spectra were determined using infrared spectroscopy (VERTEX 80V, Bruker, MA, USA).

### 2.2. Adsorption Kinetics of CIP by BNPs

To determine the optimal BNP dosage, adsorption experiments were conducted. Five BNP concentration gradients (0.04, 0.1, 0.2, 0.4, and 1 g L^−1^) were added to CIP solutions of 5 and 10 mg L^−1^ at pH = 5.5. The mixtures were placed in a shaker set at 25 °C and 200 rpm to ensure thorough mixing and a constant temperature throughout the experiment. After 24 h, supernatant samples were collected and filtered through 0.2 μm membrane filters, and the residual CIP content was determined using a UV spectrophotometer (UV-6100, Shanghai Mapada Instruments Co., Ltd., Shanghai, China) at 277 nm. Each treatment was replicated three times. Considering removal efficiency and economic factors, a dosage of 0.2 g L^−1^ was selected for the plant experiments, achieving a CIP absorption rate exceeding 80%.

### 2.3. Plant Experiment

Rice seeds (*Oryza sativa* L. var. Nanjing 9108) were obtained from the Jiangsu Academy of Agricultural Sciences Seed Station in May 2024. The seeds were sterilized with 3% (*v*/*v*) H_2_O_2_ solution for 30 min, rinsed, and soaked in deionized water for 24 h. Germination was initiated by placing the seeds on water-moistened filter paper in seedling trays at 25 °C in darkness. After 7 days, the rice seedlings were transplanted to black plastic pots containing Hoagland nutrient solution (pH 5.5) and cultivated for an additional 14 days until reaching a height of 10–13 cm. Growth conditions were maintained at 25 °C with 16 h of light at 20 °C and 8 h of darkness at 75% relative humidity. The plant samples were observed and compared on days 1, 3, 5, 7, and 9. The CIP content in roots and shoots, biomass, and endogenous hormone concentrations were measured. Six treatments were conducted: control (CK), CK + 0.2 g L^−1^ BNPs (CK + BNPs), 5 mg L^−1^ CIP (5 CIP), 5 mg L^−1^ CIP + 0.2 g L^−1^ BNPs (5 CIP + BNPs), 10 mg L^−1^ CIP (10 CIP), and 10 mg L^−1^ CIP + 0.2 g L^−1^ BNPs (10 CIP + BNPs). Each treatment was replicated three times. In a previous study, we found that BNPs alone showed no significant differences in growth parameters or hormone levels compared to untreated rice seedlings [20], and the BNP-only treatment was not performed in the following study.

### 2.4. CIP Determination

The method for extracting CIP from plant samples was adapted from Shen et al. (2020) with minor modifications [21]. Collected rice seedlings were separated into above-ground and below-ground portions. Approximately 1 g (fresh weight) samples of rice leaves were chopped, homogenized, and placed in centrifuge tubes with 10 mL of an acetone/dichloromethane (1:1 *v*/*v*) mixture. Ultrasonic extraction was performed for 30 min and repeated three times. The extract was filtered through anhydrous sodium sulfate silica gel columns and collected in 50 mL conical flasks. The columns were then washed with 10 mL of hexane/dichloromethane (1:1 *v*/*v*) mixture. The combined extracts were evaporated to dryness at 40 °C using a vacuum rotary evaporator. The residue was dissolved in 2 mL of HPLC-grade methanol, filtered through a 0.22 μm membrane, and analyzed by HPLC. CIP concentrations were analyzed using a high-performance liquid chromatography system (Thermo Fisher Scientific U3000, Waltham, MA, USA) equipped with a UV detector and an Agilent Zorbax SB-C18 column (250 nm × 4.6 nm × 5 μm). The mobile phase consisted of 20% acetonitrile and 80% water, with a column temperature of 30 °C, a flow rate of 1.0 mL min^−1^, and an injection volume of 10 μL. The UV detection wavelength for CIP was set at 279 nm. The CIP content was calculated using a standard curve method (r^2^ > 0.99).

### 2.5. Detection of Plant Endogenous Hormones

Endogenous hormones in rice seedling leaves were analyzed using an Agilent 1290 high-performance liquid chromatography system coupled with an AB SCIEX-6500Qtrap mass spectrometer following a modified method from Pan et al. (2010) [22]. The target hormones were indole-3-acetic acid (IAA), 6-benzylaminopurine (6-BA), salicylic acid (SA), jasmonic acid (JA), and abscisic acid (ABA). Fresh rice seedling leaves (approximately 50 mg) were ground in liquid nitrogen and extracted with 1 mL of extraction solution (isopropanol: ultrapure water: hydrochloric acid, 1000:500:1, *v*/*v*/*v*). Samples were shaken at 4 °C in darkness for 30 min, followed by the addition of 2 mL of dichloromethane and further shaking for 30 min. After centrifugation (13,000 rpm, 5 min, 4 °C), the lower organic phase was processed, concentrated, and reconstituted in 0.2 mL of 0.1% formic acid in methanol for HPLC-MS/MS analysis.

The hormone content was calculated using the following formula:

Hormone content (ng g^−1^) = C × V/M (1)

where C is the concentration of each component in the test solution (ng mL^−1^), V is the volume of the sample extract (mL), and M is the sample mass (g).

### 2.6. Laboratory QA/QC Protocols and Statistical Analysis

Calibration standards (0–500 ng g^−1^) and quality control samples (10, 100, 500 ng g^−1^) were prepared using a blank rice leaf matrix for the plant hormone analysis. Isotopically labeled internal standards were added to all samples, standards, and QCs before extraction. The HPLC-MS/MS method was validated for linearity (R^2^ > 0.999), accuracy (85–115% recovery), precision (<15% RSD), and sensitivity. The limits of detection (LOD) and quantification (LOQ) were estimated based on signal-to-noise ratios of 3 and 10, respectively. Quality control measures included analysis of blanks (unspiked negative controls and solvent controls), use of internal standards, analysis of duplicates, determination of spike recovery, and analysis of matrix standards. The recoveries of spiked plant samples (100 μg g^−1^) were 91.2 ± 3.0% (*n* = 3). Each treatment consisted of three replicates to calculate the mean and standard error. The hormone content was calculated using the internal standard method and expressed as ng g^−1^ fresh weight. This comprehensive QA/QC protocol ensured reliable qualitative and quantitative analysis of the target plant hormones in the rice seedling leaves.

Quality controls were conducted to ensure reliable qualitative and quantitative analysis of CIP in this study. The recoveries of CIP in spiked plant samples (100 μg g^−1^) were 91.2 ± 3.0% (n = 3). Five-point standard calibration curves were used to quantify the CIP concentrations in the solution (R^2^ > 0.999) and plant samples (R^2^ > 0.999). LOD and LOQ were estimated based on signal-to-noise ratios of 3 and 10, respectively. The LOD and LOQ for CIP were calculated to be 1.2 μg L^−1^ and 4.0 μg L^−1^ in the solution and 59.8 ng g^−1^ and 199 ng g^−1^ in the plant samples, respectively.

All statistical analyses were conducted using IBM SPSS Statistics 25 (SPSS Inc., Chicago, IL, USA). One-way ANOVA was employed to assess significant differences at a *p* < 0.05 probability level, followed by Duncan’s multiple range tests.

## 3. Results

### 3.1. Properties of BNPs and the Optimal Dosage Selection

**Figure 2:**
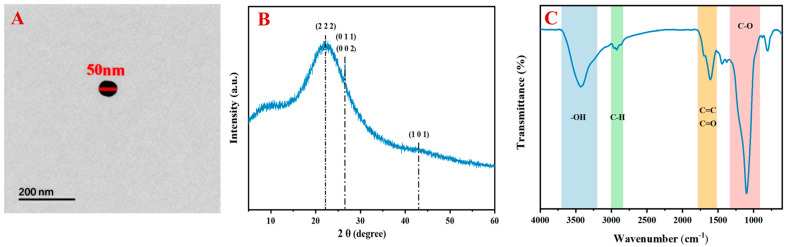
Characterizations of biochar nanoparticles. (Note, (**A**) TEM image; (**B**) XPS whole pattern; and (**C**) FTIR, respectively).

Physicochemical characterization of the BNPs synthesized via pyrolysis of corn straw at 450 °C elucidated its morphological, structural, and surface properties. Transmission electron microscopy analysis revealed discrete nanoparticles with a mean diameter of 50 ± 5 nm (Figure 2A). X-ray diffraction patterns exhibited a prominent broad peak in the low-angle region (5°–30°), indicative of a predominantly amorphous carbon structure (Figure 2B). Characteristic diffraction peaks at 2θ = 22.20°, 26.56°, and 43.08° were indexed to the (222), (002)/(011), and (101) crystallographic planes, respectively, suggesting the presence of graphitic domains within the amorphous matrix. Fourier transform infrared spectroscopy provided evidence of the following diverse surface functionalities: aliphatic C-H stretching vibrations (2836–3000 cm^−1^), hydroxyl and C-O stretching modes (3203–3702 cm^−1^ and 916–1342 cm^−1^), and aromatic C=C and carbonyl C=O vibrations (1529–1778 cm^−1^) (Figure 2C).

**Figure 3:**
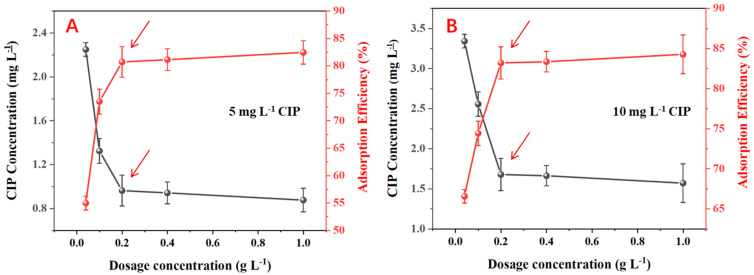
Determination of optimal dosage for biochar nanoparticles (BNPs) in ciprofloxacin (CIP) solutions. (Note, (**A**) adsorption efficiency and CIP concentration at 5 mg L^−1^ CIP; (**B**) adsorption efficiency and CIP concentration at 10 mg L^−1^ CIP. The arrows indicate the optimal BNP dosage of 0.2 g L^−1^ for both CIP concentrations).

The optimal dosage of corn straw BNPs for CIP adsorption was investigated at CIP concentrations of 5 mg L^−1^ and 10 mg L^−1^ (Figure 3). For 5 mg L^−1^ CIP, the adsorption efficiency increased rapidly from 54.8% to 80.8% as the BNPs dosage increased from 0 to 0.2 g L^−1^, with a corresponding decrease in CIP concentration from 2.26 to 0.96 mg L^−1^ (Figure 3A). Further increases in the BNP dosage beyond 0.2 g L^−1^ resulted in only marginal improvements in adsorption efficiency, reaching 82.1% at 1.0 g L^−1^ BNPs. Similarly, for 10 mg L^−1^ CIP, the adsorption efficiency rose sharply from 65.9% to 82.4% as the BNP dosage increased from 0 to 0.2 g L^−1^, with the CIP concentration decreasing from 3.41 to 1.76 mg L^−1^ (Figure 3B). Subsequent increases in the BNP dosage yielded minimal additional adsorption, achieving 84.3% efficiency at 1.0 g L^−1^ BNPs. These results indicate that 0.2 g L^−1^ was the optimal dosage of BNPs for efficient CIP adsorption at both concentrations, balancing high adsorption efficiency with economic use of the adsorbent.

### 3.2. The Phenotype Response and Growth Condition

**Figure 4:**
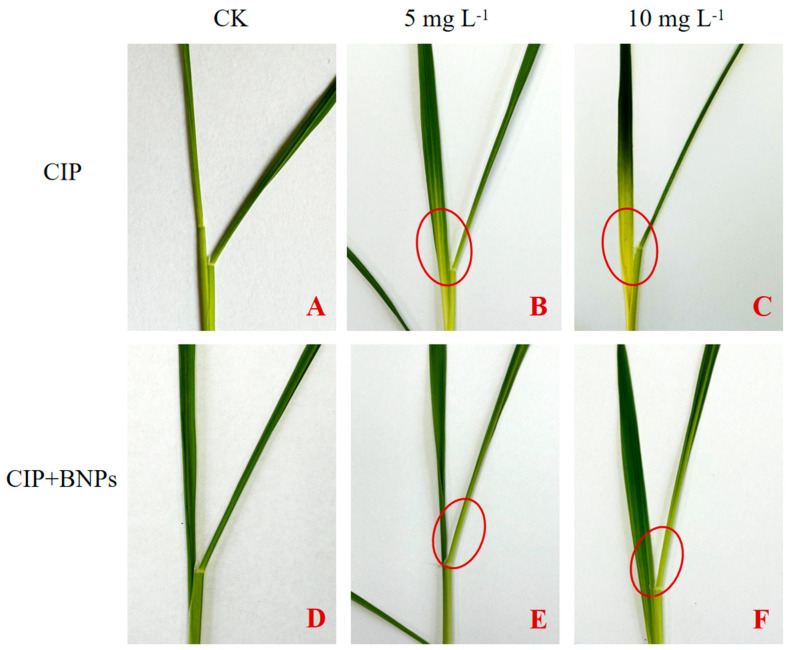
Changes in leaf phenotypes of rice seedlings after the exposure of ciprofloxacin (CIP) and biochar nanoparticles (BNPs) on day 9. Red circles indicate the discolored part of the rice seedlings’ leaf base. (Note, (**A**–**F**) leaf seedling phenotypes on day 9: (**A**) CK; (**B**) 5 mg L^−1^ CIP; (**C**) 10 mg L^−1^ CIP; (**D**) CK + 2.0 mg L^−1^ BNPs; (**E**) 5 mg L^−1^ CIP + 2.0 mg L^−1^ BNPs; (**F**) 10 mg L^−1^ CIP + 2.0 mg L^−1^ BNPs; CIP, ciprofloxacin; BNPs, biochar nanoparticles).

The phenotypic responses of the rice seedlings to CIP exposure and the influence of the BNP treatment were visually assessed and recorded, as shown in Figure 4. This visual assessment of seedling phenotypes provides insights into the potential stress-mitigating properties of BNPs in the context of CIP exposure. The most prominent phenotypic change observed was chlorosis, manifesting as yellowing at the base of the leaves in the CIP-treated seedlings. The control seedlings (Figure 4A) and those treated with BNPs alone (Figure 4D) maintained a healthy green coloration throughout their leaves, indicating normal development. CIP exposure induced distinct morphological alterations, characterized by chlorosis initiating at the leaf base and progressing acropetally. This symptom exhibited concentration-dependent severity, with the 10 mg L^−1^ CIP treatment showing more extensive chlorotic regions compared to the 5 mg L^−1^ treatment (Figure 4B,C). This chlorosis appeared more pronounced with increasing the CIP concentration from 5 mg L^−1^ to 10 mg L^−1^, suggesting a dose-dependent effect on the seedling phenotype. The plants treated with BNPs maintained an 85% greater chlorophyll-containing leaf area compared to the CIP-only treatments (Figure 4E,F). While some discoloration was still evident, particularly at higher CIP concentrations, the extent of chlorosis was reduced compared to the seedlings treated with CIP alone. The 5 mg L^−1^ CIP + BNPs treatment (Figure 4E) showed the least severe leaf discoloration among the CIP-exposed groups. These phenotypic observations, as clearly depicted in Figure 4, indicate that CIP exposure negatively impacts rice seedling development, with the severity of visible stress symptoms correlating with the CIP concentration. The addition of BNPs appears to ameliorate these adverse effects, as evidenced by the reduced chlorosis in the BNP-supplemented treatments.

**Figure 5:**
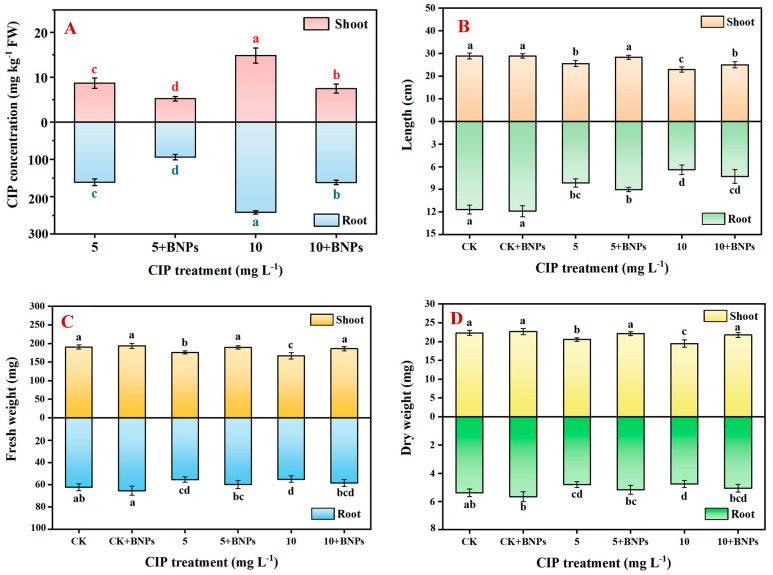
Effects of ciprofloxacin (CIP) and BNP exposure on rice seedlings. (Note, (**A**) CIP accumulation after 9 days of CIP and BNP exposure; (**B**) length of shoots and roots after 9 days of CIP and BNP exposure; (**C**) fresh weight of shoots and roots after 9 days of CIP and BNP exposure; and (**D**) dry weight of shoots and roots after 9 days of CIP and BNP exposure. BNPs, biochar nanoparticles. Data points and error bars represent means ± S.D., respectively; different letters indicate significant differences (*p* < 0.05)).

It was observed that the CIP and BNPs treatments significantly affected the shoot length at day 9 (Figure 5B). The control seedlings had a shoot length of 28.85 cm. CIP exposure significantly reduced the shoot length in a dose-dependent manner, with 5 mg L^−1^ CIP resulting in 25.52 cm (*p* < 0.05) and 10 mg L^−1^ CIP further decreasing it to 22.93 cm (*p* < 0.01). The addition of BNPs significantly mitigated this effect, with the 5 mg L^−1^ CIP + BNPs treatment restoring the shoot length to 28.33 cm, statistically similar to the control (*p* > 0.05). Compared to CIP alone, the 10 mg L^−1^ CIP + BNPs treatment significantly improved the shoot length to 24.98 cm (*p* < 0.05), showing partial recovery. Root length changes across treatments are illustrated in Figure 5B. The control root length was 11.68 cm at day 9. CIP dramatically reduced the root length, with 5 mg L^−1^ CIP resulting in 8.15 cm (*p* < 0.05) and 10 mg L^−1^ CIP further decreasing it to 6.40 cm (*p* < 0.05). The addition of BNPs provided significant partial mitigation, with root lengths of 9.03 cm and 7.30 cm for the 5 mg L^−1^ CIP + BNPs and 10 mg L^−1^ CIP + BNPs treatments (*p* < 0.05), respectively.

The shoot and root dry weight variations after 9 days of treatment are depicted in Figure 5D. The control shoot dry weight was 22.29 mg. CIP exposure significantly reduced this to 20.55 mg at 5 mg L^−1^ (*p* < 0.05) and 19.47 ± 0.99 mg at 10 mg L^−1^ (*p* < 0.05). BNP supplementation significantly ameliorated the CIP-induced reduction in shoot dry weight. The 5 mg L^−1^ CIP + BNPs and 10 mg L^−1^ CIP + BNPs treatments resulted in shoot dry weights of 22.14 mg and 21.75 mg, respectively, which were statistically indistinguishable from the control and significantly higher than the CIP-only treatments. This indicates a complete restoration of shoot biomass accumulation in the presence of BNPs, even under CIP stress conditions. The control root dry weight was 5.39 mg. CIP exposure resulted in a significant reduction in biomass of 10.9% (*p* < 0.05) at 5 mg L^−1^ and 11.7% (*p* < 0.05) at 10 mg L^−1^, respectively. The addition of BNPs provided significant partial mitigation, with root dry weights of 5.17 mg and 5.06 mg for the 5 mg L^−1^ CIP + BNPs and 10 mg L^−1^ CIP + BNPs treatments (*p* < 0.05), respectively.

### 3.3. The Hormone Response

**Figure 6:**
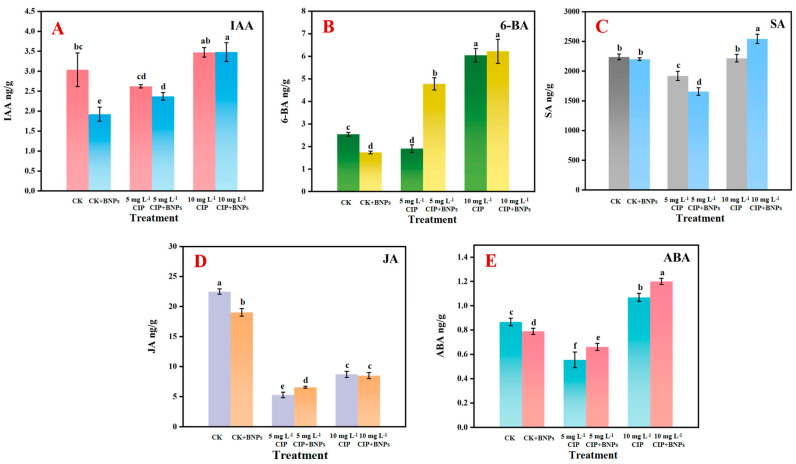
Effects of ciprofloxacin (CIP) and biochar nanoparticles (BNPs) on hormone concentrations in rice plants measured on day 9. (Note, (**A**) indole-3-acetic acid (IAA), (**B**) 6-benzylaminopurine (6-BA), (**C**) salicylic acid (SA), (**D**) jasmonic acid (JA), and (**E**) abscisic acid (ABA). Data points and error bars represent means ± S.D., respectively. Different letters indicate significant differences (*p* < 0.05) between treatments).

The effects of the CIP and BNP treatments on the rice plant hormone levels were assessed on day 9 (Figure 6). The effects of the treatments on indole-3-acetic acid (IAA) levels were examined (Figure 6A). CIP exposure at 5 mg L^−1^ resulted in a significant decrease in IAA (14.5%, *p* < 0.05), while 10 mg L^−1^ CIP induced a significant increase of 15.8% (*p* < 0.05) relative to the control. BNP supplementation mitigated these effects, elevating IAA levels by 8.9% and 2.9% in the 5 and 10 mg L^−1^ CIP treatments, respectively (*p* < 0.05). These results indicate a non-linear dose response to CIP and a modulating effect of the BNPs on IAA concentrations. The impact on the 6-benzylaminopurine (6-BA) concentrations was notable (Figure 6B). CIP at 5 mg L^−1^ significantly reduced 6-BA by 28.6% (*p* < 0.05), whereas 10 mg L^−1^ CIP markedly increased 6-BA by 126.7% (*p* < 0.05) compared to the control. The addition of BNPs significantly altered the 6-BA levels, resulting in a 152.6% increase with the 5 mg L^−1^ CIP + BNPs treatment and a 3.3% increase with the 10 mg L^−1^ CIP + BNPs treatment relative to CIP alone (*p* < 0.05). These findings demonstrate a pronounced dose-dependent effect of CIP and a substantial modulating influence of BNPs on 6-BA levels. Alterations in salicylic acid (SA) concentrations were also observed (Figure 6C). The CIP treatment induced dose-dependent decreases in SA, with reductions of 15.9% and 32.7% at 5 and 10 mg L^−1^ CIP, respectively (*p* < 0.05). BNP supplementation partially ameliorated these effects, increasing SA levels by 12.7% in the 5 mg L^−1^ CIP + BNPs treatment (*p* < 0.05) and by 13.6% in the 10 mg L^−1^ CIP + BNPs treatment (*p* < 0.01) compared to CIP alone. Notably, BNPs elevated SA levels above the control values at higher CIP concentrations, suggesting a compensatory mechanism. Jasmonic acid (JA) concentrations were significantly affected by the treatments (Figure 6D). CIP exposure significantly diminished JA levels, with reductions of 72.9% and 60.4% observed at 5 and 10 mg L^−1^ CIP, respectively (*p* < 0.05). The addition of BNPs partially counteracted this effect, increasing JA levels by 20.0% and 2.2% in the 5 and 10 mg L^−1^ CIP + BNPs treatments, respectively, compared to CIP alone (*p* < 0.05). However, JA concentrations did not fully recover to the control levels, indicating a persistent impact of CIP exposure. In addition, changes in abscisic acid (ABA) concentrations were also observed, as shown in Figure 6E. CIP at 5 mg L^−1^ significantly decreased ABA levels by 34.5% (*p* < 0.05), while 10 mg L^−1^ CIP increased ABA levels by 20.2% above the control levels (*p* < 0.05). BNP supplementation further elevated ABA levels, resulting in increases of 18.2% and 12.3% in the 5 and 10 mg L^−1^ CIP + BNPs treatments, respectively, compared to CIP alone (*p* < 0.05). The highest ABA concentration was observed in the 10 mg L^−1^ CIP + BNPs treatment, suggesting a potential synergistic effect at higher CIP concentrations.

## 4. Discussion

### 4.1. The Effect of CIP on Hormone and Growth Regulation

Based on the above results, CIP exposure significantly altered the hormonal balance in the Oryza sativa seedlings, which subsequently impacted growth parameters. The complex interplay between CIP, plant hormones, and growth regulation can be elucidated as follows. CIP induced dose-dependent and non-linear changes in various phytohormones (Figure 6). At 5 mg L^−1^, CIP decreased IAA levels by 14.5%, while at 10 mg L^−1^, it increased IAA levels by 15.8% (Figure 6A). This biphasic response suggests a hormetic effect of CIP on auxin metabolism [1]. Similarly, 6-benzylaminopurine (6-BA) levels showed a dramatic shift from a 28.6% decrease at 5 mg L^−1^ CIP to a 126.7% increase at 10 mg L^−1^ CIP (Figure 6B), indicating a complex regulation of cytokinin biosynthesis or degradation under CIP stress [23]. Jasmonic acid levels were severely reduced by 72.9% and 60.4% at 5 and 10 mg L^−1^ CIP (Figure 6D), respectively, while ABA levels showed a concentration-dependent response, decreasing by 34.5% at 5 mg L^−1^ CIP but increasing by 20.2% at 10 mg L^−1^ CIP (Figure 6E). These CIP-induced hormonal imbalances correlated strongly with the observed growth inhibitions. The reduction in IAA levels at lower CIP concentrations likely contributed to the decreased shoot length of 11.5% at 5 mg L^−1^ CIP, given auxin’s crucial role in cell elongation and division [24]. The severe reduction in JA levels across both CIP concentrations may explain the significant inhibition of root growth of 30.2% and 45.2% at 5 and 10 mg L^−1^ CIP, respectively, due to the role of JA in regulating root development [25]. The increase in ABA levels at higher CIP concentrations could further exacerbate growth inhibition, particularly in roots, as ABA is a known growth suppressor under stress conditions [26]. The non-linear responses of hormones to CIP exposure suggest complex interactions between the antibiotic and hormone biosynthesis or signaling pathways. For instance, the contrasting effects of low and high CIP concentrations on IAA and 6-BA levels indicate that CIP may interfere with different aspects of hormone metabolism, depending on its concentration [27]. The consistent suppression of JA levels across the CIP concentrations suggests that CIP might directly or indirectly inhibit JA biosynthesis enzymes or upregulate JA catabolism [28]. The dose-dependent effect on ABA levels implies that CIP may modulate ABA biosynthesis or catabolism differently at various concentrations, possibly through interaction with enzymes involved in the ABA metabolic pathway [29]. The CIP-induced hormonal changes collectively contribute to the observed growth inhibitions. The reduction in shoot and root biomass (Figure 4C,D) can be attributed to the combined effects of decreased auxin at lower CIP concentrations, suppressed JA, and elevated ABA at higher CIP concentrations [30]. The complex changes in cytokinin levels may further complicate growth responses, as cytokinins play crucial roles in cell division and organ development [31]. The interplay between these hormonal changes likely disrupts the delicate balance required for optimal growth, resulting in the observed phenotypic alterations (Figure 4). It is suggested that CIP exposure induces a complex network of hormonal changes in rice seedlings, which, in turn, lead to significant growth inhibitions. The non-linear and dose-dependent nature of these hormone-mediated effects highlights the intricate interactions between CIP and plant physiological processes. Understanding these mechanisms is crucial for developing strategies to mitigate CIP-induced phytotoxicity in agricultural systems.

### 4.2. Adsorption Properties and Direct CIP Removal

The application of BNPs demonstrated remarkable efficacy in mitigating CIP-induced stress effects on the rice seedlings. The adsorption mechanism is primarily governed by their unique physicochemical properties. Transmission electron microscopy revealed discrete nanoparticles with a mean diameter of 50 nm (Figure 2A), providing a high surface-area-to-volume ratio that enhances adsorption capacity. This observation aligns with Wang et al. (2022), who reported that smaller biochar particles exhibited higher adsorption capacities for antibiotics [32]. The X-ray diffraction patterns indicated a predominantly amorphous carbon structure with graphitic domains (Figure 2B), likely contributing to the BNPs’ ability to interact with organic contaminants through π-π electron donor–acceptor interactions [33].

The FTIR analysis revealed diverse surface functionalities, including hydroxyl, carbonyl, and aromatic groups (Figure 2C), which interact with CIP molecules through (1) electrostatic interactions between the negatively charged BNP surfaces and zwitterionic CIP molecules [34,35]; (2) hydrogen bonding between the hydroxyl groups of BNPs and the carboxyl/amine groups of CIP [36,37]; and (3) π-π stacking interactions between aromatic structures [38,39]. The effectiveness of these mechanisms was demonstrated by the optimal adsorption achieved at a level of 0.2 g L^−1^ BNPs for both CIP concentrations (Figure 2).

### 4.3. Physiological Response Modulation and Growth Recovery

BNP supplementation significantly ameliorated CIP-induced reductions in plant growth. In the 5 mg L^−1^ CIP + BNPs treatment, the shoot length was restored to 98.2% of the control, while the root length recovered to 77.3%, compared to 88.5% and 69.8%, respectively, with CIP alone. This growth recovery correlated with the partial restoration of IAA levels and a significant increase in 6-BA concentrations (Figure 6A,B).

Beyond direct CIP adsorption, BNPs influenced hormone homeostasis by altering the rhizosphere environment, affecting nutrient availability, microbial activity, and root-to-shoot signaling pathways [40]. Studies have confirmed that nanoparticles can enter plant cells through nanoscale pores [41] and transport via the xylem or phloem [42,43]. BNPs influence gene expression in plant cells [44,45], affecting hormone biosynthesis, transport, and signaling cascades [46].

The elevation of SA levels above the control values at higher CIP concentrations (Figure 6C) indicates the activation of systemic defense mechanisms, similar to the observation of Kumar et al. (2021) in biochar-treated tomato plants [47]. The partial restoration of JA levels suggests a role of BNPs in modulating stress-related hormones through the octadecanoid pathway (Figure 6D). Additionally, BNPs showed synergistic effects on ABA levels at higher CIP concentrations (Figure 6E), potentially influencing ABA metabolism through key enzymes like NCED or ABA 8′-hydroxylase, consistent with the findings of Langeroodi et al. (2019) [48].

The stress reduction mechanisms are further evidenced by the following: (1) reduced phytotoxicity through high CIP adsorption and enhanced nutrient dynamics, and (2) improved photosynthetic efficiency, as shown by reduced chlorosis (Figure 4) and enhanced chloroplast protection. These multi-faceted protection mechanisms, combining direct contaminant adsorption with the modulation of physiological responses, offer a promising strategy for mitigating antibiotic stress in agricultural systems.

## 5. Conclusions

This study demonstrates the effective mitigation of CIP-induced stress in rice seedlings by BNPs through multiple quantifiable mechanisms. The mitigation effects are evidenced by the following: (1) a direct reduction in CIP bioavailability, with BNPs achieving >80% adsorption efficiency at 0.2 g L^−1^, resulting in 49.7% and 33.1% decreased CIP accumulation in shoots and roots, respectively; (2) a restoration of growth parameters, where BNPs mitigated CIP-induced growth inhibition by recovering the shoot length to 98.2% of the control levels at 5 mg L^−1^ CIP and improving root development from 69.8% to 77.3%; and (3) a reestablishment of hormone balance, with a significant recovery in the levels of IAA (+8.9%), 6-BA (152.6% increase), and stress-responsive hormones (12.7–13.6% improvement in SA levels).

The physicochemical characteristics of BNPs, including a 50 nm mean particle diameter and diverse surface functionalities, enabled these mitigation effects through efficient CIP adsorption. These findings advance our understanding of using biochar nanoparticles to mitigate antibiotic stress in agricultural systems. Future research should focus on optimizing BNPs for enhanced stress reduction across different soil conditions and crop species.

## Data Availability

Data will be made available on request.
